# The Importance of Effective Organizational Socialization for Preventing Stress, Strain, and Early Career Burnout: An Intensive Longitudinal Study of New Professionals

**DOI:** 10.3390/ijerph19127356

**Published:** 2022-06-15

**Authors:** Elin Frögéli, Stefan Annell, Ann Rudman, Miguel Inzunza, Petter Gustavsson

**Affiliations:** 1 Department of Clinical Neuroscience, Karolinska Institutet, 17177 Stockholm, Sweden; ard@du.se (A.R.); petter.gustavsson@ki.se (P.G.); 2 Department of Leadership and Command & Control, Swedish Defence University, 11428 Stockholm, Sweden; stefan.annell@fhs.se; 3 Department of Caring Sciences, School of Health and Welfare, Dalarna University, 79131 Falun, Sweden; 4 Police Education and Research Unit, Umeå University, 90187 Umeå, Sweden; miguel.inzunza@umu.se

**Keywords:** burnout, intensive longitudinal design, JD-R, new professionals, onboarding, organizational socialization, strain, stress, transition

## Abstract

Burnout was originally conceptualized based on experiences of new professionals. Role clarity, task mastery, and social acceptance are recognized as key resources enabling new professionals’ management of the challenges of the new profession. However, relations between these resources and stress, strain, and burnout have not yet been thoroughly investigated at professional entry. Increased understanding of these relations could have implications for strategies to prevent burnout. The aim of the study was to investigate within- and between-individual effects over the first months and relations to burnout at one-year post-entry. Data (*n* = 322) was collected weekly over the first 13 weeks and again 9 months later. Relationships were modelled using a multilevel regression model and correlation analysis. Results showed that on weeks when participants experienced higher role clarity, task mastery, and social acceptance, they reported significantly less stress, and that participants who experienced higher levels of the resources in general, reported significantly less strain. Levels of the resources at three months were related to symptoms of burnout at 12 months. The study findings provide support of the role of task mastery, role clarity, and social acceptance as resources buffering the impact of demands at professional entry on experiences of stress, strain, and burnout.

## 1. Introduction

In Europe, every forth worker experience that their health is at risk due to conditions of working life [1]. In Sweden, stress-related mental ill-health, including the clinical conceptualization of burnout referred to as exhaustion disorder, is the major cause of long-term sick-leave [2]. Burnout is defined as “a persistent, negative, work-related state of mind in ‘normal’ individuals that is primarily characterized by exhaustion, which is accompanied by distress, a sense of reduced effectiveness, decreased motivation, and the development of dysfunctional attitudes and behaviors at work” ([3], p. 36).

One of the leading models of burnout is the job demands–resource (JD-R) model [4]. The model posits that burnout is the result of exposure to job demands in combination with inadequate resources. Job demands are those physical, psychological, social, or organizational aspects of work that requires sustained effort and are therefore, over time, associated with increased risks of certain physical and/or psychological costs (strain) and development of symptoms of burnout. Job resources are those physical, psychological, social, or organizational aspects of work that are functional in achieving work goals and stimulating personal growth, learning, and development. A specific form of resources are personal resources that refers to peoples’ acquired beliefs about their abilities to exert influence over their environment. One of the factors assumed to contribute to the popularity of the JD-R model is the fact that it is not occupation-specific but rather may flexibly be applied to different occupations [4].

Resources (including acquired personal resources) are assumed to have a buffering role on the impact of job demands on the risk of developing symptoms of burnout [4]. Put differently, a lack of resources constitutes a risk factor for developing symptoms of burnout. One period in working life that is particularly characterized by high demands in combination with a lack of resources is the transition from education to working life [5]. Since the original conceptualizations of burnout, the vulnerability of new professionals has been acknowledged [6,7]. Population health data in Sweden show that young adults (16–29 years) are the population group with the highest prevalence of symptoms of stress, anxiety, and reduced mental well-being [8]. Young adults (24–35 years) are also the population group with the highest levels of sick leave due to stress-related mental ill-health [2]. 

Today, new professionals’ experiences in encountering working life are typically studied within the framework of organizational socialization [9]. According to this framework, the most important resources for new professionals in managing the challenges of entering a new professional role are task mastery, role clarity, and social acceptance [5]. Task mastery concerns the experiences of being able to manage tasks effectively and affects the degree to which situations in the new profession are perceived as controllable. Role clarity concerns the newcomers’ knowledge of what is expected within their new professional role and what level of influence they may exert and affects the degree to which situations in the new profession are perceived as predictable. Finally, social acceptance concerns the new professionals’ inclusion into their new group of colleagues and affects the degree to which new professionals experience social risks. 

New professionals who experience high stress and symptoms of burnout at professional entry report lower levels of job satisfaction [10] and higher levels of turnover intentions [11]. In addition, individuals who experience symptoms of burnout during the first years of working life experience more cognitive problems and sleep problems a decade later as compared to individuals who do not experience the same symptoms at the start of their careers, corrected for ongoing symptoms [12]. 

However, there has been a lack of guidance in terms of how to prevent experiences of stress and burnout among new professionals [13]. Combining the propositions of the JD-R model with organizational socialization research suggests that organizations who wish to reduce the risk of burnout among their new employees should ensure that they experience high levels of role clarity, task mastery, and social acceptance. This is also in line with the propositions of a model of socialization through the lens of the newcomer stress appraisal process [13]. However, with the exception of one study showing that development of role clarity, task mastery, and social acceptance was related to decreased levels of stress week-by-week, as well as lower levels of strain over the first three months following professional entry among newly registered nurses [14], the effects of the development of these resources during the first months of professional life on ongoing experiences of stress, strain, and the risk of developing symptoms of burnout are understudied within organizational socialization research [13]. In addition, there is still a need for more investigations of the job demands–personal resources interaction [4]. Therefore, more research investigating these relations among new professionals within other professional groups, confirming or disconfirming previous findings, is needed to better understand the problem of burnout among new professionals and to identify or develop preventative strategies. In this study, we include a number of professions that have received attention recently.

### 1.1. Aims

The aim of the present study was to investigate how perceived role clarity, task mastery, and social acceptance relate to experiences of stress and strain during the first 13 weeks following professional entry as well as the risk of developing symptoms of burnout nine months later in a heterogeneous sample of new professionals, all working in occupations characterized by high emotional demands (e.g., teachers, social workers, and police officers).

### 1.2. Hypotheses

**Hypothesis** **1** **(H1).** *On weeks when participants experience higher levels of role clarity, task mastery, and social acceptance as compared to their own mean over time, they will report lower levels of stress (within-person effect interpreted as indication of an effect of the acquired personal resources on stress)*.

**Hypothesis** **2** **(H2).** *Participants who experience a higher level of role clarity, task mastery, and social acceptance as compared to the sample mean over the first 13 weeks in the profession will report lower levels of stress (between-persons effect interpreted as indication of an effect of the acquired personal resources on strain)*.

**Hypothesis** **3** **(H3).** *New professionals who experience a higher level of role clarity, task mastery, and social acceptance three months into the new profession will report lower levels of burnout one year following professional entry (correlations between the acquired personal resources and burnout symptoms interpreted as an indication of and effect of adjustment on the risk of developing symptoms of burnout)*.

## 2. Materials and Methods

### 2.1. Study Design

In this study, we used an intensive longitudinal design [15] with weekly data collections over a period of 13 weeks following professional entry as well as follow-up data from one year into the profession. Ethical approval for the study was granted by the Swedish Ethical Review Authority (document number 2017:543-31).

### 2.2. Setting, Participants, and Recruitment

The study was conducted in Sweden during the spring and summer of 2018. Eligible participants were students who were about to graduate and start their first employment as lawyers, physicians, nurses, teachers, social workers, police officers, and military officers during the period of the study. Participants were recruited from 12 universities. Information about the study was presented verbally and in text by a representative from the research group at an information meeting at each university. Information about the study was also made available through university-platforms and emails, as well as a Facebook page. Interest to participate in the study was registered through a digital platform. A signed, completed, and submitted baseline questionnaire was interpreted as informed consent to participate in the study.

### 2.3. Variables

#### 2.3.1. Outcomes

Stress was measured using an adapted version of the Stress and Energy questionnaire (SEQ [16]). The adapted version included three items asking about the frequencies of experiences of being stressed, pressured, or tense during the last week at work. The items were responded to using a five-point Likert scale ranging from ‘Never’ to ‘Many times per day’. The internal consistency reliability (between-persons reliability) was 0.94, and the reliability of change (within-person reliability) was 0.78, as estimated by omega coefficients from a multi-level confirmatory factor analysis [15]. 

Burnout was measured using the Shirom Melamed Burnout Measure (SMBM [17]). The measure consists of 14 items asking about symptoms of burnout in the three categories mental exhaustion, cognitive fatigue, and social fatigue. The items were responded to using a five-point Likert scale ranging from ‘Very often or always’ to ‘Very seldom or never’. Cronbach’s alpha for the data included in this study was 0.95.

#### 2.3.2. Predictors

Three measures were included to represent the acquired personal resources. Role clarity was measured using a three-item short-version of the General Questionnaire for Psychological and Social Factors at Work (QPS-Nordic [18]). The items asked about how often during the last week the new professionals had experienced their professional role (including demands and responsibilities) to be clear to them. The internal consistency reliability was 0.93 and the reliability of change was 0.70, as estimated by omega coefficients from a multi-level confirmatory factor analysis [15].

Task mastery was measured using two adapted items from the Needs Satisfaction and Frustration Scale (NSFS [19]). The two items asked about how often during the last week the new professionals had experienced that they were able to perform even the most challenging tasks, as well as how often they had experienced that they had performed well at their job. The internal consistency reliability was 0.95 and the reliability of change was 0.66, as estimated by omega coefficients from a multi-level confirmatory factor analysis [15].

Finally, social acceptance was measured using two items from the Needs Satisfaction and Frustration Scale (NSFS [19]). The two items asked about how often during the last week the new professionals had experienced that their co-workers cared about them, as well as how often they had experienced that they were included in the group of colleagues. The internal consistency reliability was 0.93 and the reliability of change was 0.72, as estimated by omega coefficients from a multi-level confirmatory factor analysis [15].

All predictor items were responded to using a five-point Likert-scale ranging from 1 (‘Very seldom or never’) to 5 (‘Very often or always’). The instruments measuring the outcome as well as the predictors in this study have previously been used to monitor stress levels and acquired personal resources among new professionals using an intensive longitudinal study design [14].

### 2.4. Data Collection

Data were collected using a digital survey [20]. During the first 13 weeks following professional entry, at the same time and day of every week of the study, an email was sent out to participants including an individualized URL through which they gained access to the survey questions. Remainder emails were sent to participants who had not responded to the survey within four days. Each survey was active for one week. Data on experiences of stress were collected every week of the study. Data on the role clarity, task mastery, and social acceptance were collected from the second week. One year following professional entry, using the same digital survey and method, a follow-up survey was sent to participants to collect data on experiences of symptoms of burnout. 

### 2.5. Sample Size Calculation

There is a lack of rules of thumb regarding the sample size needed for intensive multilevel regression. However, in [14], statistically significant relations between perceived role clarity, task mastery, social acceptance, and stress in the sizes of −0.33 to −0.40 (within-person) and −0.57 to −0.77 (between-person) were detected with a sample of 264 new nurses. Accordingly, a sample of 300 new professionals was assumed to be large enough for the present study.

### 2.6. Statistical Methods

Data were analyzed using a multilevel model approach for intensive longitudinal data, as suggested by [15]. For the analyses, time was rescaled to represent time in months, ranging from 0 to 3. Raw data were visualized with individual panel plots and spaghetti plots to investigate the linearity of within-person change as well as average change over time. These plots were prepared using IBM SPSS Statistics 23.

Relations between experiences of role clarity, task mastery, social acceptance, and stress as well as strain were investigated using multilevel models with the within-person and between-person versions of the variables as predictors [15]. Parameters were estimated using the default procedures for these models in Mplus 8.2, Muthén and Muthén, Los Angeles, CA, USA (Bayes estimation). A statistically significant within-person version of a predictor variable was interpreted as indicating that weekly changes in perceived role clarity, task mastery, and social acceptance were related to a short-term or acute activation/deactivation of the stress response. Furthermore, a statistically significant between-person version of a predictor variable was interpreted as indicating that the perceived level of role clarity, task mastery, and social acceptance over time was related to a strain reaction (i.e., a sustained stress response).

To model and describe the development over time in stress, role clarity, task mastery, and social acceptance, we used linear growth curve analyses with full maximum likelihood estimation [15]. We initially tested a model including a quadratic effect of time, and if no statistically significant quadratic effect could be confirmed, we tested a model with a linear effect of time only, in line with the standard procedure [21]. 

To investigate the relations between perceived role clarity, task mastery, and social acceptance three months into a new profession, and the risk of experiencing symptoms of burnout one year following professional entry, bivariate correlation analyses were performed. For each participant, the maximum level of acquired personal resources reported during the week 9–13 period of the study was entered into the analyses together with reported symptoms of burnout at one year. 

## 3. Results

### 3.1. Participants

**Figure 1:**
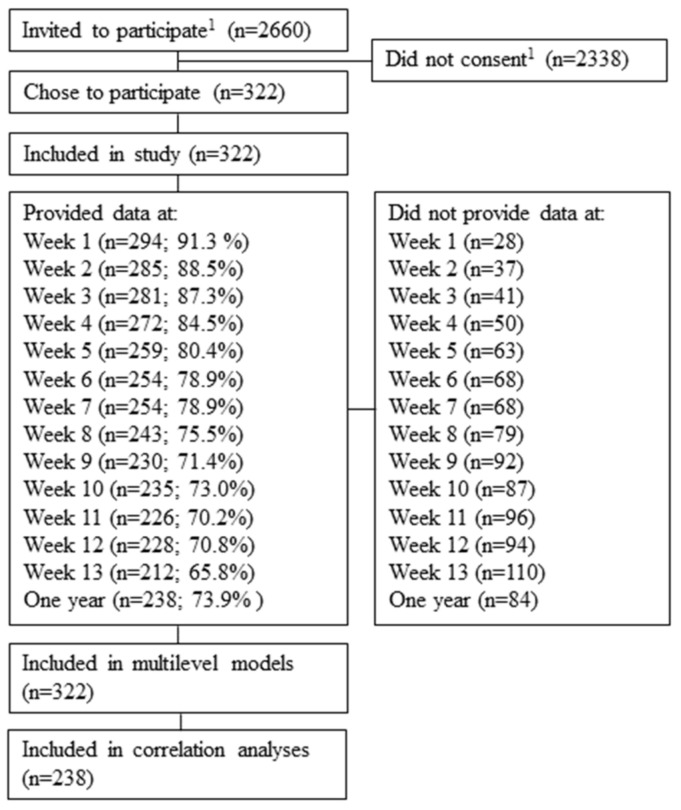
Flow of participants throughout the study. Notes: ^1^ = we do not know how many actually received the information and fulfilled the criteria of eligibility (graduating + starting to work in the upcoming weeks).

In total, 322 participants were included in the study. In Figure 1, the flow of the participants throughout the study is presented. Participation at each weekly survey (1–13) ranged from 93.1% of the total sample at the first survey to 65.8% at the last (number of respondents per survey are presented in Figure 1). The most common response pattern was to answer 13/13 surveys. One year into the profession, the survey was responded to by 73.9% of respondents. The number of respondents at each occasion of the study are presented in Table 1. Drop out could not be explained by any of the demographic variables.

### 3.2. Descriptive Data

#### 3.2.1. Characteristics of Study Participants

The median age of the participants was 28, with a range from 21 to 56. The majority were female (241; 74.8%), one forth (79; 24.5%) were males, and two participants (0.6%) registered as another alternative gender. 

Approximately two thirds (209; 65.1%) of the sample were in a relationship, whereas 112 participants (34.9%) were single. Most of the participants (227; 70.7%) reported that both of their parents were born in Sweden, but 46 (14.3%) and 47 (14.6%) of participants reported one or both of their parents were born in another country. 

The most common occupation amongst the participants was registered nurse (98; 30.4%) followed by physician (67; 20.8%). The remaining participants reported working as teachers (57; 17.7%), social workers (45; 14.0%), police officers (32; 9.9%), military officers (17; 5.3%), and lawyers (6; 1.9%). 

#### 3.2.2. Missing Data

The number of expected responses as well as actual response for each outcome measure are presented in Table 1. In addition, the percentage of actual responses are given in relation to the expected.

#### 3.2.3. Summary Measures and Development over Time

The mean and standard deviation of each study variable and week are presented in Table 2. The estimates of the linear growth models of change over time are presented in Table 3. The typical study subject experienced a linear decrease in stress over the period of the study as indicated by the statistically significant fixed effect of time. In addition, the typical subject experienced an increase in perceived role clarity and task mastery over the period of the study as indicated by the statistically significant fixed effects of time. For task mastery, a statistically significant negative quadratic effect could be confirmed, indicating that the rate of the increase decreased with the passage of time in the study. Finally, for social acceptance, there was no statistically significant change for the typical subject over the period of the study.

### 3.3. Outcome Data

#### 3.3.1. Relations to Stress and Strain during the First 13 Weeks of the Profession

On weeks when participants reported a higher perceived level of role clarity, task mastery, and social acceptance as compared to their own individual mean over time (within-person association presented in Table 4), they experienced a lower level of stress. In addition, participants who reported higher perceived levels of role clarity, task mastery, and social acceptance over time reported lower levels of strain, as indicated by the between-person associations in Table 4. All within- and between-person associations were statistically significant. The estimates of the effects were larger for task mastery than they were for role clarity and social acceptance.

#### 3.3.2. Relations to Burnout One Year following Professional Entry

Perceived levels of role clarity, task mastery, and social acceptance three months into the profession were negatively correlated with perceived symptoms of burnout one year into the profession. Social acceptance (after three months) had the largest correlation with burnout at the one-year follow-up (r = −0.29, *p* = 0.001), while the correlations for role clarity (r = −0.27, *p* = 0.001) and task mastery (r = −0.21, *p* = 0.002) was slightly lower.

## 4. Discussion

In this study, using an intensive longitudinal study design, we collected data weekly over the first 13 weeks following professional entry in a heterogeneous sample of new professionals. In addition, follow-up data were collected one year into a profession. On weeks when participants reported higher perceived levels of role clarity, task mastery, and social acceptance, they reported lower levels of stress. Furthermore, participants who perceived their role clarity, task mastery, and social acceptance to be greater over time experienced lower levels of strain in general. In addition, achieved levels of perceived role clarity, task mastery, and social acceptance three months into a profession were negatively related to perceived symptoms of burnout one year into a profession.

The present study is the second study to confirm a relationship between the three key personal resources of new professionals and stress week-by-week as well as strain over the first three months using an intensive longitudinal study design. The study addresses a request to further investigate the assumed demands–personal resources interaction proposed by the JD-R model [4] and thereby contributes to the understanding of the relationship between personal resources, demands, and the risk of experiencing stress and strain, and developing symptoms of burnout. Furthermore, this study expands the results of the previous study with a similar design [14] beyond new nurses by including a range of new professionals within other professions and by investigating relations to perceived symptoms of burnout one year into a profession. This is important, as much of the burnout literature is focused on healthcare professionals [22]. The results of this study suggest that by supporting role clarity, task mastery, and social acceptance, organizations may help to reduce stress and strain among new professionals in different occupations, as well as reduce the risk of developing symptoms of burnout. 

The term onboarding refers to “all formal and informal practices, programs, and policies enacted or engaged in by an organization or its agents to facilitate newcomer adjustment” [23]. The purpose of onboarding is to structure newcomers’ early experiences, which is expected to foster the development of their personal resources and facilitate their adjustment [23]. Results of two important meta-analyses show that higher levels of role clarity, task mastery, and social acceptance may be achieved by treating newcomers in a standardized way, within an explicit plan and time frame, and a special set of discrete, predefined experiences that they may engage in, as well as engaging experienced organizational members as role models [5,24]. The results of the present study add to these previous findings by suggesting that such onboarding practices may also contribute to reducing stress, strain, and symptoms of burnout among new professionals.

Interestingly, in this study, as well as in [14], task mastery came out as the acquired personal resources for which the relations (between-person and within-person) to stress and strain during the first three months were the strongest. This finding is hopeful and inspiring as task mastery is something that we know may be developed using structured practices. This is perhaps particularly important as the growth curve analyses in the present study showed that the increase in task mastery was particularly pronounced in the beginning of the study period, and then the pace of increase decreased. Together, these findings suggest that organizations need to be extra conscious of strategies for maintaining a positive development of task mastery beyond the initial period of adjusting to the new professional role. Specifically, [25] highlights the importance of making it possible for someone who is to learn a new skill to gain mastery experiences; that is, to experience confidence and competence in executing a task. This is also in line with previous recommendations from the organizational socialization literature suggesting that newcomers should be given on-the-job training and be encouraged to monitor how co-workers perform their tasks [23,26,27].

Achieved levels of role clarity, task mastery, and social acceptance three months into the profession were negatively related to the risk of experiencing symptoms of burnout nine months later; that is, one year into a profession. This further highlights the importance of supporting the adjustment of new professionals at professional entry, as this may be expected to have important consequences over extended periods of time. This finding is supported by a previous study showing that achieved levels of the acquired personal resources one year into the profession was related to concurrent levels of symptoms of burnout, and that development of the resources from year one to year two, as well as year one to year three in a new profession, is related to a concurrent decrease in symptoms of burnout [28].

### 4.1. Limitations

We attempted to reduce the risk of bias due to a non-representative sample by using a digital survey and recruiting participants from multiple universities in Sweden. Still, the number of graduating students who chose to participate was less than desired. Recruitment was particularly challenging in certain professional groups as well as among men. 

In our investigation of the relations between role clarity, task mastery, social acceptance and experiences of stress, strain, and symptoms of burnout, we did not consider the potential impact of other variables. It is possible that the relations differ between new professionals with different amount of prior professional experiences, different ages, gender, etc. It is also possible that results would differ between individuals with different methods of managing the stress that they are experiencing at professional entry. In a previous line of studies, we developed and tested an intervention focused on increasing new professionals’ active management of the challenges that they were encountering as well as increasing their engagement in recovery behaviors on and off work [29,30]. Using a randomized parallel group design, we found that experiences of stress were prevented in the group who participated in the experimental intervention, and that the effect was particularly pronounced among those how were more active in changing their behaviors [30]. That said, in the present study, to reduce this risk of bias due to response fatigue, we included a minimum set of items in each survey. This explains why some potentially interesting variables (such as proactive management of challenges) were excluded as well as why we did not include some of the measures typically used within organizational socialization research.

### 4.2. Conclusions

There has been a request for investigations of experiences of stress as part of organizational socialization. In the present study, we found that (1) on weeks when an individual experienced a greater role clarity, task mastery, and social acceptance as compared to his or her own mean over time, this experience was related to a decrease in experiences of stress; (2) individuals who experienced greater levels of role clarity, task mastery, and social acceptance in general experienced lower levels of strain; and (3) greater perceptions of role clarity, task mastery, and social acceptance three months into the new profession is related to lower levels of symptoms of burnout one year into a profession. These findings are in line with the assumption from organizational socialization research and the JD-R model, suggesting that supporting the development of the personal resources role clarity, task mastery, and social acceptance is one important way forward for reducing experiences of stress and symptoms of burnout among new professionals. Supporting the development of task mastery (e.g., tasks with graded difficulty, sufficient time, support, and feedback) is particularly important as the effects of perceived task mastery on experiences of stress and strain were the strongest but the rate of the development of task mastery decreased over time.

## Figures and Tables

**Table 1:** Total number of expected and actual responses week 1–13 and one year into a profession.

Variable.	Time Point	Expected Responses (*n*)	Actual Responses (*n*, % of Expected)
Stress	Week 1–13	4186	2706, 64.6%
Task mastery	Week 2–13	3864	2466, 63.8%
Role clarity	Week 2–13	3864	2465, 63.8%
Social acceptance	Week 2–13	3864	2467, 63.8%
Burnout	One year	322	202, 62.7%

Note: *n* = number.

**Table 2:** Summary measures (means and standard deviations) of study variables week 1–13 (*n* = 212–294) and one year into a profession (*n* = 238).

Time Point	Stress M (SD)	Task Mastery M (SD)	Role Clarity M (SD)	Social Acceptance M (SD)	Burnout M (SD)
Week 1	2.60 (1.05)				
Week 2	2.78 (1.08)	3.57 (0.91)	3.69 (0.78)	4.14 (0.84)	
Week 3	2.75 (1.00)	3.64 (0.84)	3.67 (0.79)	4.10 (0.83)	
Week 4	2.77 (1.05)	3.71 (0.80)	3.80 (0.68)	4.14 (0.81)	
Week 5	2.73 (1.01)	3.72 (0.88)	3.79 (0.75)	4.18 (0.86)	
Week 6	2.72 (1.06)	3.69 (0.84)	3.70 (0.81)	4.12 (0.88)	
Week 7	2.71 (1.03)	3.71 (0.91)	3.88 (0.72)	4.16 (0.85)	
Week 8	2.74 (1.12)	3.72 (0.83)	3.83 (0.79)	4.13 (0.88)	
Week 9	2.65 (1.09)	3.68 (0.88)	3.62 (0.81)	4.04 (0.86)	
Week 10	2.60 (1.10)	3.70 (0.89)	3.79 (0.75)	4.12 (0.89)	
Week 11	2.57 (1.12)	3.71 (0.90)	3.84 (0.75)	4.09 (0.90)	
Week 12	2.57 (1.03)	3.74 (0.86)	3.68 (0.81)	4.08 (0.89)	
Week 13	2.60 (1.08)	3.65 (0.90)	3.83 (0.69)	4.07 (0.92)	
One year	-	-	-	-	2.42 (0.87)

Note: M = mean; SD = standard deviation.

**Table 3:** Linear growth curve models showing development over time (*n* = 322).

Variable	Effect	Parameter	Est. (Post. SD)	*p*	95% CI	
Stress	Fixed	I	2.76 (0.06)	0.001	2.65	2.88
		T	−0.05 (0.02)	0.008	−0.10	−0.01
	Random	I	0.83 (0.08)	0.001	0.69	1.01
		T	0.07 (0.01)	0.001	0.05	0.10
		I × T	−0.05 (0.02)	0.014	−0.09	−0.01
Role clarity	Fixed	I	3.72 (0.04)	0.001	3.63	3.80
		T	0.04 (0.02)	0.006	0.01	0.07
	Random	I	0.34 (0.04)	0.001	0.31	0.47
		T	0.03 (0.01)	0.001	0.02	0.04
		I × T	−0.02 (0.01)	0.041	−0.04	0.00
Task mastery	Fixed	I	3.62 (0.05)	0.001	3.51	3.72
		T	0.13 (0.06)	0.011	0.02	0.24
		T^2^	−0.04 (0.02)	0.022	−0.07	−0.00
	Random	I	0.56 (0.07)	0.001	0.45	0.72
		T	0.30 (0.08)	0.001	0.16	0.47
		Q	0.03 (0.01)	0.001	0.02	0.05
		I × T	−0.17 (0.06)	0.001	−0.29	−0.07
		I × Q	0.04 (0.02)	0.003	0.01	0.08
		T × Q	−0.09 (0.02)	0.001	−0.14	−0.04
Social acc.	Fixed	I	4.14 (0.05)	0.001	−0.09	−0.02
		T	−0.02 (0.02)	0.146	−0.06	0.02
	Random	I	0.50 (0.05)	0.001	0.41	0.61
		T	0.07 (0.01)	0.001	0.05	0.09
		I × T	−0.05 (0.02)	0.001	−0.09	−0.02

Note: I = intercept; T = time; Q = T^2^; Est. = parameter estimate; post. (SD) = posterior standard deviation; *p* = probability value; CI = confidence interval; Social acc. = social acceptance.

**Table 4:** Fixed effects of relations between between-person and within-person components of role clarity, task mastery, and social acceptance, and perceived stress (*n* = 322).

Variable	Parameter							
	Between-Person				Within-Person			
	Est. (Post. SD)	*p*	95% CI		Est. (Post. SD)	*p*	95% CI	
Role clarity	−0.51 (0.08)	0.001	−0.65	−0.33	−0.18 (0.03)	0.001	−0.25	−0.13
Task mastery	−0.63 (0.06)	0.001	−0.75	−0.50	−0.25 (0.04)	0.001	−0.30	−0.15
Social acc.	−0.41 (0.07)	0.001	−0.54	−0.22	−0.13 (0.03)	0.001	−0.19	−0.07

Note: social acc. = social acceptance; Est = parameter estimate; post. (SD) = posterior standard deviation; *p* = probability value; CI = confidence interval; Social acc. = social acceptance.

## Data Availability

The data that support the findings of this study are available from the corresponding author upon reasonable request.
